# Expression of B-class MADS-box genes in response to variations in photoperiod is associated with chasmogamous and cleistogamous flower development in *Viola philippica*

**DOI:** 10.1186/s12870-016-0832-2

**Published:** 2016-07-07

**Authors:** Qiaoxia Li, Qingdi Huo, Juan Wang, Jing Zhao, Kun Sun, Chaoying He

**Affiliations:** Life Science College, Northwest Normal University, Anning East Road 967, Anning, 730070 Lanzhou, Gansu China; State Key Laboratory of Systematic and Evolutionary Botany, Institute of Botany, Chinese Academy of Sciences, Nanxincun 20, Xiangshan 100093 Beijing, China

**Keywords:** Adaptive evolution, Dimorphic flower, Gene expression, MADS-box gene, Photoperiod, *Viola philippica*

## Abstract

**Background:**

Some plants develop a breeding system that produces both chasmogamous (CH) and cleistogamous (CL) flowers. However, the underlying molecular mechanism remains elusive.

**Results:**

In the present study, we observed that *Viola philippica* develops CH flowers with short daylight, whereas an extended photoperiod induces the formation of intermediate CL and CL flowers. In response to long daylight, the respective number and size of petals and stamens was lower and smaller than those of normally developed CH flowers, and a minimum of 14-h light induced complete CL flowers that had no petals but developed two stamens of reduced fertility. The floral ABC model indicates that B-class MADS-box genes largely influence the development of the affected two-whorl floral organs; therefore, we focused on characterizing these genes in *V. philippica* to understand this particular developmental transition. Three such genes were isolated and respectively designated as *VpTM6-1*, *VpTM6-2*, and *VpPI*. These were differentially expressed during floral development (particularly in petals and stamens) and the highest level of expression was observed in CH flowers; significantly low levels were detected in intermediate CL flowers, and the lowest level in CL flowers. The observed variations in the levels of expression after floral induction and organogenesis apparently occurred in response to variations in photoperiod.

**Conclusions:**

Therefore, inhibition of the development of petals and stamens might be due to the downregulation of B-class MADS-box gene expression by long daylight, thereby inducing the generation of CL flowers. Our work contributes to the understanding of the adaptive evolutionary formation of dimorphic flowers in plants.

**Electronic supplementary material:**

The online version of this article (doi:10.1186/s12870-016-0832-2) contains supplementary material, which is available to authorized users.

## Background

Flowers are typically composed of four organ types: sepals, petals, stamens, and carpels, which run from the outside of the flower to the center. The ABC model of flower development explains how three major function class genes (A-, B-, and C-class) specify the identity of the four floral organ types. A-class alone controls sepals, A-class in combination with B-class controls petals, B-class in combination with C-class controls stamens, and C-class alone controls carpels [1, 2]. A pair of MADS-box genes, *APETALA3* (*AP3*) and *PISTILLATA* (*PI*) in *Arabidopsis thaliana*, and *DEFICIENS* (*DEF*) and *GLOBOSA* (*GLO*) in *Antirrhinum majus*, encodes B-function activity [3–6]. Mutations in either the *AP3/DEF* or *PI*/*GLO* genes results in similar phenotypic variations, wherein petals are transformed into sepals, and stamens into carpels [7–9]. The B-class lineages apparently underwent duplications and subsequent functional divergence in some core eudicots, possibly playing a role in the diversification of floral morphology during evolution [10–16]. For example, in Solanaceae and Leguminosae, the *PI* lineage duplicated into two *GLO*-like genes (*GLO1* and *GLO2*), and the *AP3* lineage underwent a duplication event at the base of the core eudicots, giving rise to two *AP3*-like lineages called the *euAP3* and *paleoAP3* genes [14–20]. The *euAP3* genes include *AP3/DEF*, and the *paleoAP3* type genes were named *TOMATO MADS BOX GENE 6* (*TM6*) genes, after the first isolated member from *Solanum lycopersicum* [21]. Although these paralogous genes are partially redundant, they have largely contributed to the development of stamens and petals. Interestingly, the *TM6* lineage was subsequently lost in *Arabidopsis* and *Antirrhinum* [22]. In contrast, the *AP3* lineage was lost in papaya, which now only contains *TM6* [23]. These results indicate that it is possible to retain either or both of the *AP3/TM6* paralogous pair and still produce flowers [22].

B-class MADS-box genes are not only involved in the specification of organ identity of petals and stamens, but also in the control of organ maturation. Knocking down *AP3/PI* at intermediate stages (stages 8–10) in *Arabidopsis* flowers induces petal-to-sepal transformations that gradually occur in consecutive buds. However, although stamens in these flowers retain their identity, they become increasingly underdeveloped and do not dehisce pollen [24]. Independent petal loss within the buttercup family (Ranunculaceae) is strongly associated with decreased or eliminated expression of a single floral organ identity gene, *AP3-3* [25]. In rice, the B-class MADS-box gene, *SUPERWOMAN1* (*SPW1*), specifies the identities of lodicules (equivalent to petals) and stamens. This was supported by the observation in the mutant of this gene, *superwoman1-cleistogamy* (*spw1-cls*), of normal stamens, where lodicules were transformed homeotically to lodicule-glume mosaic organs, thereby engendering cleistogamy [26]. The phenotypic effect observed in the downregulation of *Medicago truncatula TM6* (*MtTM6*) activity involved a change in petal shape, as well as some stamens differentiating into filaments and anthers failing to produce pollen grains [14]. Furthermore, severely downregulating either *Physalis floridana GLO2* (*PFGLO2*) or *Physalis floridana TM6* (*PFTM6*) only results in poor pollen maturation [15, 16].

Several plants, including most species in the genus *Viola* within the family Violaceae are dimorphic cleistogamy plants capable of producing both open potential outcross and closed selfing flowers [27]. Two examples are such as *Viola odorata* [28, 29] and *V. pubescens* [30]. The open flowers are also called chasmogamous (CH) flowers, while the closed flowers called cleistogamous (CL) flowers*.* CL flowers may be energetically less costly to produce, resulting in more resources available for seed production and disruption of locally adapted gene complexes. On the other hand, CH flowers produce genetically variable progeny favored in spatially or temporally heterogeneous habitats [31–33]. Therefore, the evolution of dimorphic flowers at a single event exhibits the highest fitness when each flower type is produced in the environment for which it is best suited [27]*.* Compared to CH flowers, CL petals are undeveloped and functional stamens become smaller and decreased in number. Moreover, *V. odorata* CH flowers are formed in response to short daylight, whereas CL flowers emerge in response to long daylight [28, 29]. In *V. pubescens*, orthologous genes for gibberellins 20 oxidase (*VGA20ox*) and gibberellins 3 oxidase (*VGA3ox*) are upregulated in CH flowers compared to that in CL flowers, thereby indicating a role for gibberellins (GA) in the differential production of flower types [34]. However, the molecular mechanisms underlying this developmental transition are largely unknown in *Viola*. In the present study, we revealed the variations in the development of petals and stamens, which are the major affected floral organs in the formation of dimorphic flowers in *V. philippica* under different photoperiods. Furthermore, we investigated the potential role of B-class MADS-box genes in the development of the CH-CL breeding system. Three B-class MADS-box genes were cloned and designated as *VpTM6-1*, *VpTM6-2*, and *VpPI*. We determined that the differential expression of these genes during late floral development under the different photoperiods was correlated to the development of CH and CL flowers, thus providing novel insights into the development and evolution of dimorphic flowers in plants.

## Results

### Different types of floral morphology in *V. philippica*

**Fig. 1:**
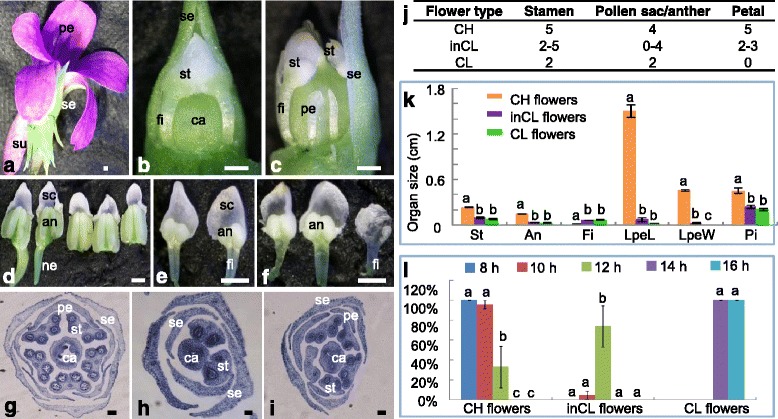
Floral morphological variations under different photoperiods in *V. philippica*. **a** CH flower. **b** CL flower. **c** inCL flower. **d** CH flower stamens. Five stamens with invisible filament develop, showing distinct nectar glands. **e** CL flower stamens. Only two stamens with visible filament develop. **f** inCL flower stamens. Three to five stamens develop with visible filaments. g-**i** The cross sections of flowers. **g** CH flowers. Five stamens and five petals are observed. Each stamen has four pollen sacs. **h** CL flowers. Two stamens and no petal are detected. Each stamen has two pollen sacs. **i** inCL flowers. Two to three petals, and two to five stamens develop in these flowers. Each stamen has zero to four pollen sacs. The microsporogenesis in some stamens is inhibited. Bars = 500 um (**a**–**f**). Bars = 200 um (**g** and **i**), 100 um (**h**). se, sepal; pe, petal; su, spur; st, stamen; ca, carpel; an, anther; sc, stamen cap; fi, filament; ne, nectar gland. **j** Variations in the number of floral organs. **k** Variations in floral organ size. The length of stamens (St), anthers (An), filaments (Fi), lower petals (LpeL), and pistils (Pi), and the width of lower petals (LpeW) are measured. **l** The CH-CL floral transition under different photoperiods. Standard errors are provided and lower-case letters (a, b, and c) indicate significant differences (*P* < 0.05)

*V. philippica* could develop both CH and CL flowers, although the CL flowers were approximately five to six times smaller than CH flowers (Fig. 1). CH flowers had five large and showy petals, with the lowest protruding slightly at the base into a spur, and five stamens forming a cone surrounding the pistil. Each stamen had four pollen sacs, and the lowest two stamens had noticeable nectar glands attached to them (Fig. 1a, d, g, and j). CL flowers had two stamens without nectar glands, each stamen had two pollen sacs, and the five petals were all undeveloped (Fig. 1b, e, h, and j). In certain conditions, intermediate CL (inCL) flowers developed, and they displayed variable characteristics. Typical observations were: between one and three poorly developed petals, two to five developed stamens, with each stamen having two to four pollen sacs but no nectar glands (Fig. 1c, f, i, and j). In extreme cases, a few poorly developed stamens in inCL flowers had one pollen sac, or even no pollen sac and just a membranous appendage (Fig. 1i and j). In addition, we found that the stamen length, anther length, pistil length, lower petal length, and width of CH flowers were all significantly greater than those of CL and inCL flowers. Conversely, the filament of CL and inCL stamens was distinct compared to that of CH flowers, which was undetectable (Fig. 1d, e, f, and k). No homeosis of floral organs was observed in the CH-CL transition.

### Photoperiods affect flower development in *V. philippica*

Under natural conditions, *V. philippica* produced complete CH flowers in the early spring and a mixture of CH and inCL flowers in late spring and late autumn, whereas complete CL flowers developed in the summer and early autumn, suggesting that dimorphic flower development in *V. philippica* might be regulated by photoperiods. We thus set five photoperiods (8-, 10-, 12-, 14-, and 16-h daylight) to test this assumption. Results showed that three types of flowers developed under these conditions. Complete CL flowers were formed under long-day lights (14- and 16-h daylight), and CH flowers uniquely developed in 8-h daylight. At 10–12-h daylight, both CH and inCL flowers simultaneously developed and > 90 % of the floral buds were CH flowers under a photoperiod of 10-h daylight. The CH/inCL + CLratio significantly decreased with longer photoperiods (Fig. 1l), indicating that CL flower development was induced by long-day light. Therefore, different photoperiods may affect the development of petals and stamens, thus regulating the formation of different types of flower morphology in *V. philippica*. To further understand this formation, we next examined the floral organ initiation process.

### Organ initiation and development of different flower types in *V. philippica*

**Fig. 2:**
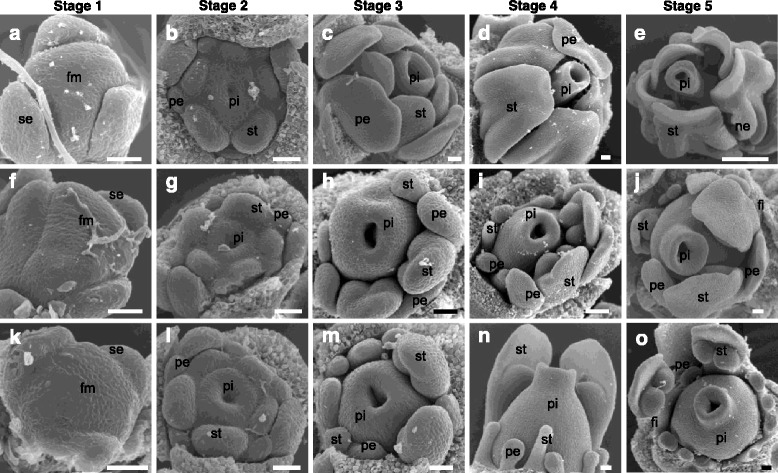
Floral initiation, organogenesis, and development in *V. philippica*. **a**–**e** CH flowers. **f**–**j** inCL flowers. **k**–**o** CL flowers. Floral induction and development as revealed by scanning electron microscopy (SEM) analysis, and five stages (Stage 1 to 5 as indicated) are roughly defined. se, sepal; pe, petal; st, stamen; ne, nectar; pi, pistil; fi, filament; fm, flower meristem. Bars = 50 um (**a**–**d**, **f**–**j**, **k**–**o**), and 500 um (**e**)

*V. philippica* is a perennial plant with a (2 + 3) phyllotaxis shoot system, similar to that in *V. odorata* [28]. Roughly five floral developmental stages were defined based on the position of phyllotaxis using a series of landmark events and observation with a scanning electron microscope. In general, the floral buds closer to the central tender leaf were determined to be relatively young. We comparatively depicted the floral development of CH flowers that developed under short daylights, as well as CL and inCL flowers that developed under long and transitional daylights. The first stage involved the generation of the floral meristem, whereas the second stage showed floral organogenesis. Four whorl floral organ primordia of CH, as well as inCL and CL flowers, were observed in the floral meristem, indicating no obvious differences in the first and second stages in these three types of flowers (Fig. 2a, b, f, g, k, and l). However, at the third stage, after four whorl organ primordia formation, CH flowers had five obvious petals and stamens, whereas CL flowers had two stamens, and other stamens and all petals only consisted of organ primordial structures (Fig. 2c and m). There were two to five stamens, and at least lower petals that were poorly developed in inCL flowers (Fig. 2h). At the fourth stage (Fig. 2d, i, and n), four whorl floral organs of CH flowers continued to develop, and the style was higher than the stamens. The style of the CL and inCL flowers began to bend to the two fully developed stamens. At the fifth stage (Fig. 2e, j, and o), the nectar glands at the base of the two stamens began to appear in the CH flowers. The lower petals, side petals, or other stamens in the inCL flowers were poorly developed to some extent and later became more distinct, whereas five petals and the other three stamens (except for two big stamens) remained in the organ primordial state in the CL flowers (Fig. 2j and o). The filaments of CL and inCL stamens became relatively distinct (Fig. 2j and o). The development of the three stamens and all petals were then completely arrested, and these floral organs remained as primordia in the CL flowers, which developed under long daylight. These comparative observations suggested that early floral development was indistinguishable between CH and CL flowers, whereas the morphological divergence leading to the dimorphic flowers occurred at stage 3. This divergence was mainly attributable to the arrest of petal and stamen development in response to the extension of the photoperiod. Because the growth and differentiation of both petals and stamens were to some extent inhibited in the CH-inCL-CL floral transition in *V. philippica* with extended photoperiods, we next investigated the role of B-class MADS-box genes in the development of these two-whorl floral organs.

### Isolation and sequencing analysis of B-class MADS-box genes in *V. philippica*

**Fig. 3:**
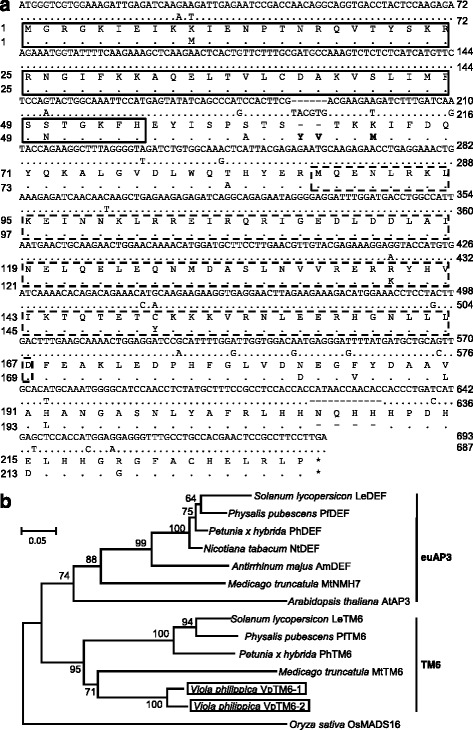
Molecular characterization of *AP3*-like MADS-box genes in *V. philippica*. **a** The ORFs of the *VpTM6* genes and its putative peptides. Gaps are introduced to show indels between the two paralogs. The sequences of *VpTM6-1* are presented, and the identical sequences in *VpTM6-2* are highlighted using dots. The star indicates the stop codon. The M, I, K, and C domains of the hypothetic VpTM6 proteins are indicated. The M domain is highlighted by solid boxes, and the K domain is indicated by dashed boxes. The region between M and K domains is the I domain, while the C domain is behind the K domain. The deleterious amino acid substitutions were highlighted in bold (for details, see Additional file 2: Table S2). **b** Neighbor-joining (NJ) tree of *AP3*-like genes. *VpTM6-1* and *VpTM6-1* are boxed. The accession numbers of other sequences used are *LeTM6* (X60759.1), *LeAP3* (DQ674532.1), *PhTM6* (AF230704.1), *PhDEF* (X69946.1), *MtTM6* (JN412097.1), *MtNMH7* (JN412096.1), *AtAP3* (NM115294.5), *AmDEF* (AB516402.1), *NtDEF* (X96428.1), *PfDEF* (KC174703.1), and *PfTM6* (KC174704.1). *OsMADS16* (AF077760.1) is used as outgroup. The species name is listed before the gene name. The numbers next to the nodes are bootstrap values. Multiple sequence alignment for the NJ tree is shown in Additional file 3: (Figure S1a)

Using a combination of homologous fragment amplification and rapid amplification of cDNA ends (RACE), we acquired the cDNA of the three genes as putative members of both *AP3*- and *PI*-lineages of the MADS-box genes. These two lineage genes usually feature a paleoAP3, euAP3, or PI motif at the end of the C-terminal region [17]. BLAST analysis identified two *V. philippica AP3* paralogs that shared the highest sequence identity (66.8 % and 64.6 %) at the amino acid level with putative *Petunia* TM6 proteins (Additional file 1: Table S1), indicating that these were *TM6*-like genes, and were thus designated as *VpTM6-1* and *VpTM6-2.* The open reading frame (ORF) of the *VpTM6-1* and *VpTM6-2* gene was 693 and 687 bp in length, respectively, and encoded predicted polypeptides of 230 and 228 amino acids in length, respectively. Indels and single nucleotide polymorphisms (SNPs), including 41 nucleotide alteration positions were observed between these two cDNA sequences that resulted in 19 amino acid substitutions (Fig. 3a). Furthermore, these two paralogs shared 92.6 % sequence identity at the amino acid level (Additional file 1: Table S1). The four domains (M, I, K and C) of MADS-domain proteins play an essential role in DNA-binding and protein-protein interaction [35, 36]. Distribution of 2 ~ 10 amino acid substitutions, including deletions, was observed in each domain of the hypothetical VpTM6 proteins (Fig. 3a). Most of the amino acid substitutions in VpTM6-2, compared to VpTM6-1, were of different properties, and two of these (K66M, and S63_T64insYV) were predicated to be deleterious in function (Fig. 3a; Additional file 2: Table S2). These divergences thereby suggested functional divergence of the two duplicates. Multiple sequence alignment of these two genes, together with representatives of some previously functionally inferred *AP3*-like genes, showed that these two *AP3* paralogous genes had a *paleoAP3* motif instead of an *euAP3* motif at the C-terminal end (Additional file 3: Figure S1a), further suggesting that these were putative *TM6* orthologs. Phylogenetic analyses using the neighbor-joining (NJ) method further showed that these two *AP3* paralogous genes were grouped together with previously reported *TM6*-like genes, such as the closely homologous genes from tomato, *Petunia*, and *Physalis* (Fig. 3b). These analyses clearly confirmed that the isolated two *V. philippica AP3* lineage genes were *TM6* orthologs. Unfortunately, *AP3*-lineage genes with the euAP3 motif were not isolated in the present study.

**Fig. 4:**
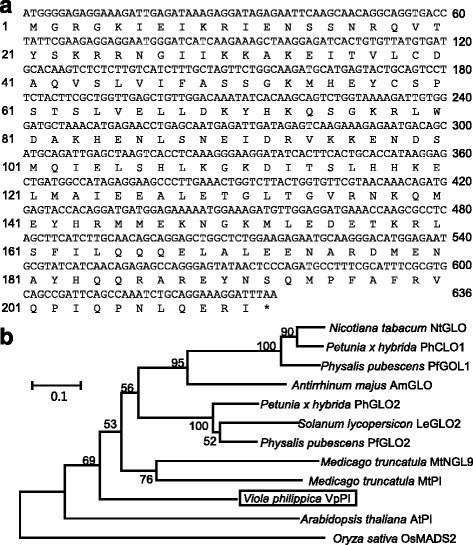
Molecular characterization of *PI*-like MADS-box genes in *V. philippica*. **a** The ORF of *VpPI* and its putative peptide. The star indicates the stop codon. **b** MP tree of *PI*-like genes. *VpPI* is boxed. The accession numbers of other sequences used are *NtGLO* (X67959.1), *PhGLO1* (M91190.1), *PhGLO2* (X69947.1), *PfGLO1* (JX467691.1), *PfGLO2* (KC174706.1), *AmGLO* (AB516403.1), *LeGLO2* (DQ674531.1), *MtNGL9* (FJ403469.1), *MtPI* (FJ403468.1), and *AtPI* (NM122031.3). *OsMADS2* (L37526.1) is used as outgroup. The species name is listed before the gene name. The numbers next to the nodes are bootstrap values. Multiple sequence alignment for the NJ tree is shown in Additional file 3: (Figure S1b)

BLAST analysis also indicated that we isolated a *PI* putative ortholog in *V. philippica*, which we designated as *VpPI*. The ORF of the *VpPI* was 636 bp in length, and its cDNA encoded a predicted polypeptide of 211 amino acids in length (Fig. 4a) that was highly similar to *Medicago PI* (71.3 % amino acid identity) and other PI orthologs from various plant species (Additional file 1: Table S1). Multiple sequence alignment showed that the amino acid sequence of the *V. philippica PI*-homolog (*VpPI*) clearly had the *PI* motif at the C-terminal region (Additional file 3: Figure S1b). Reconstruction of a NJ tree showed *VpPI* clustering with *PI* (Fig. 4b). Therefore, *VpTM6-1*, *VpTM6-2,* and *VpPI* belonged to the homologous sequences of the B-class MADS-box genes.

### Expression of *VpTM6* and *VpPI* during floral induction and organogenesis

Were the poorly developed or undeveloped petals and stamens of inCL and CL flowers in *V. philippica* related to variations in the expression of B-class MADS-box genes? To answer this question, we first examined the spatial and temporal patterns of expression during floral organogenesis and development of each flower type using in situ hybridization. Since the sequences of *VpTM6-1* and *VpTM6-2* were highly similar, a probe designed from *VpTM6-2* was used in in situ hybridization to detect the expression of these two paralogs (designated as *VpTM6*). Results showed that *VpTM6* and *VpPI* were continuously expressed from early stage (the second stage) to the later stage (the fifth stage) of floral organogenesis and development*.* At the floral organogenesis stage, *VpTM6* was not only expressed in the primordia that normally developed into petals, stamens and pistils in CH, inCL, and CL flowers, but also in the primordia that either generated poorly developed petals and stamens or were retarded (Additional file 4: Figure S2a–l). In addition, in the later development stage of floral buds, *VpTM6* was not only expressed in the developed petals and stamens in CH, inCL, and CL flowers, but also in the poorly developed or undeveloped petals and stamens of inCL and CL flowers. *VpPI* shared a similar expression domain as *VpTM6* during floral organogenesis and development of CH, inCL, and CL flowers (Additional file 4: Figure S2m–x). Sense probes did not show any hybridization signals (Additional file 4: Figure S2y). We expected to observe distinct differences in the levels of expression of these genes between the fully developed and poorly or undeveloped floral organs in correlation to the three different types of flowers. However, no obvious difference in the spatial and temporal patterns of B-class gene expression was observed during floral organogenesis and development under any test conditions. The reason underlying these unexpected observations may be attributable to the decreased suitability of an in situ hybridization technique for quantification. Therefore, we investigated the expression of *VpTM6-1*, *VpTM6-2,* and *VpPI* using qRT-PCR.

### Late floral expression of *VpTM6* and *VpPI* in response to various photoperiods

**Fig. 5:**
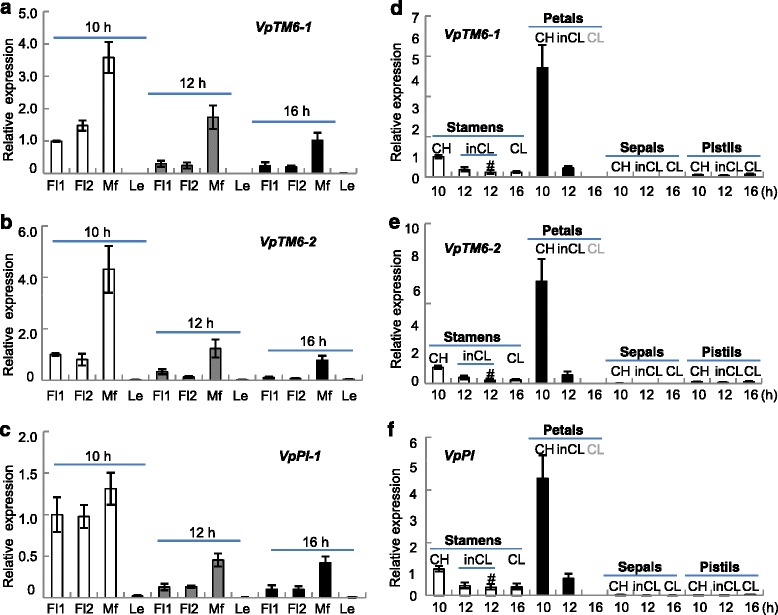
The floral expression of B-class MADS-box genes in response to variations in photoperiod. **a**–**c** Relative expression of *VpTM6-1, VpTM6-2*, and *VpPI* in flower buds and mature flowers. **a** *VpTM6-1.* **b** *VpTM6-2*. **c** *VpPI*. Gene expression in flower buds around the third stage (Fl1), the fourth to fifth stages (Fl2), mature flowers (Mf) and leaves (Le) under 10- (open columns), 12- (gray columns) and 16-h (black columns) daylight were evaluated using qRT-PCR. The gene expression in Fl1 under 10-h daylight was set to 1. **d**–**f** Relative expression of B-class MADS-box genes in floral organs of mature flowers. **d** *VpTM6-1.* **e** *VpTM6-2*. **f** *VpPI*. Gene expression in stamens (open columns), petals (black columns), and pistils (gray columns) from CH, inCL, and CL flowers under the indicated photoperiods were measured using qRT-PCR. No visible expression signal was detected in sepals under various conditions. # indicates poorly developed stamens in inCL flowers. Gray “CL” indicates that the petal was not collected from the CL flowers. The level of gene expression in the stamens of CH flowers subjected to 10-h daylight was set to 1. Three independent biological samples were used in all these analyses. Mean and standard errors are presented

To reveal the potential role of gene expression variation in response to photoperiods, we chose CH, inCL, and CL flower buds that corresponded to three photoperiods (10-, 12-, and 16-h daylight) for expression study. Two types of flower buds were used: the stage 3 flower buds (Fl1) that started to show distinct differences in morphology among CH, inCL, and CL flowers; and the stage 4 to 5 flower buds, which were designated as Fl2. Mature flowers (Mf) and leaves were also included. Using Fl1 of the CH flowers as control (Fig. 5a–c), we determined that *VpTM6-1*, *VpTM6-2*, and *VpPI* were expressed at extremely low levels in leaves (Fig. 5a–c). In contrast, these genes were mainly expressed in floral tissues, and had a tendency to increase during flower development under all conditions (Fig. 5a–c). Moreover, *VpTM6-1* and *VpTM6-2* in each floral tissue under 10-h daylight were all higher than in those subjected to 12- and 16-h daylight (Fig. 5a and b), thereby suggesting that the level of expression of *VpTM6-1* and *VpTM6-2* decreased as the duration of daylight was extended. A similar pattern of expression was observed in *VpPI* (Fig. 5c). These results indicated that the expression of the isolated MADS-box genes was predominantly expressed in floral organs, and hinted that the expression of these genes during late floral development stages might be regulated by photoperiods.

To investigate further, we examined the expression levels of *VpTM6-1*, *VpTM6-2*, and *VpPI* in floral organs of mature flowers (Fig. 5d–f). The floral organs, including sepals, petals, stamens, and pistils, corresponding to different photoperiods, were collected from CH, inCL, and CL flowers. No expression of *VpTM6-1* (Fig. 5d), *VpTM6-2* (Fig. 5e), and *VpPI* (Fig. 5f) was detected in the sepals of all flower type, and expressed at a relatively low level in all the pistils. In contrast, these were highly expressed in the petals and stamens of all flowers (Fig. 5d–f). We also observed that the level of expression of *VpTM6-1*, *VpTM6-2,* and *VpPI* in the stamens and petals of the CH flowers were much higher than in the corresponding organs of inCL and CL flowers (Fig. 5d–f). In particular, the level of expression of these genes significantly decreased in poorly developed stamens and petals of inCL flowers (Fig. 5d–f).

These observations indicated that the significant decrease in the expression of B-class MADS-box genes after floral organogenesis was induced by photoperiod extension, playing an essential role in the formation of CL flowers.

### Protein-protein interactions between VpTM6s and VpPI

To distinguish the divergence of *VpTM6-1* and *VpTM6-2*, we investigated protein-protein interactions (PPI) among the three B-class MADS-domain proteins using a yeast GAL4 two-hybrid system. Both activating *His*3 and *LacZ* reporters respectively enabling cell growth of the transformed yeast and the generation of the blue coloration in the non-lethal ß-galactosidase assay were used to demonstrate PPIs. No toxicity and self-activation of these proteins in yeast were observed, and a dramatic difference in cell growth and ß-galactosidase activity in yeast (Additional file 5: Figure S3) suggested that VpTM6-1 interacted strongly with VpPI in yeast*,* whereas the interaction between VpTM6-2 and VpPI was very weak, further implying a dramatic difference of transcriptional activity of the two heterodimmers. These results suggest functional divergence of the two VpTM6 paralogs. Nonetheless, no homodimerization of these B-class MADS-domain proteins was observed (Additional file 5: Figure S3).

## Discussion

Some plants produce both CH and CL flowers in different ecological conditions during the growth cycle. Extensive studies have focused on how various ecological factors, including water, light intensity, photoperiod, soil fertility, and temperature could affect the CH-CL mixed breeding system [30, 37–39]. The general conclusion is that dimorphic flower development might be the result of adaptive evolution. The present study investigated dimorphic flower development under different photoperiods in *V. philippica*, including its underlying molecular mechanisms.

### Dimorphic flower development and photoperiod regulation

The development of floral organs could be inhibited in nature; however, during the early developmental stage of flowers, all floral organ primordia are generally formed. For example, retardation or arrest of some floral organs after initiation occurs in most unisexual plants. Dioecious plants (such as white campion and Sorrel) and monoecious species (such as cucumber and London plant tree) all go through a hermaphroditic stage early in flower development, followed by differential abortion or arrest of sex organs, which can occur at various stages [40–43]. This phenomenon has also been observed in dimorphic cleistogamy plants. CL flowers of *Lamium amplexicaule* exhibit the least number of modifications, and no significant morphological differences are observed until pollen meiosis occurs compared to its CH flowers [44]. In the present study, no morphological differences at floral induction and organogenesis were detected in *V. philippica*. However, at later flower development stages, CH flowers featured normal flower structure, while petal and partial stamen development was retarded in CL flowers. In most cases, CL flowers had no petals and only a few fertile anthers. The arrest of floral organs is ubiquitous in nature for a variety of reasons [27, 28, 30, 40–43]. Similar to observations in temperate plant species [27, 34], the phenology of the two flower types in *V. philippica* is mainly determined by photoperiod. This phenomenon was also observed in *V. odorata*, in which CH flowers were formed in response to 11-h or less of daylight, CL flowers developed in response to 14-h or more of daylight, and inCL were produced during transitional periods [28]. Therefore, we confirmed that floral organ morphologic differentiation in *V. philippica* is determined during organogenesis after floral induction, and is influenced by photoperiod in dimorphic flower plants.

### Floral expression of *VpTM6* and *VpPI* in response to variations in photoperiod

Genome-wide gene expression patterns from cross-species microarray analysis of *Cardamine kokaiensis* of Brassicaceae has suggested that a substantial amount of genes, including petal and stamen developmental genes, are expressed differentially between CH and CL flowers [45]. However, the key regulatory gene in controlling the CH-CL transition has not been identified. In the present study, the floral organs most affected in the CH-CL transition as the photoperiod was extended were the petals and stamens. The floral ABC model indicates that B-class MADS-box genes determine the development of the two types of floral organs [1, 7, 46]. Modification of these genes either in terms of sequence or expression in various plants leads to the morphological diversification of related floral organs [13, 14, 25, 47–52]. To understand the molecular switches related to this CH-CL transition, we investigated these genes in *V. philippica*. Three B-class MADS-box genes, *VpTM6-1*, *VpTM6-2,* and *VpPI* were isolated. The coding sequence variation of these B-class gene homologs does not immediately suggest any functional novelties relative to those in non-dimorphic flower model plants, such as *Arabidopsis* and *Petunia*. Nonetheless, the expression of these genes was significantly altered in CH, CL, and inCL flower development. In line with no distinguishable differences during early flower development and no observed floral homeosis, these genes shared a relatively similar expression pattern during floral organogenesis of CH, CL, and inCL flowers. However, in the later flower development stage, where distinct floral morphology (particularly the size/number of petals and stamens) were observed in CL and inCL flowers, the expression of *VpTM6-1*, *VpTM6-2,* and *VpPI* in flowers (particularly in petals and stamens) significantly decreased in CL and inCL flowers, especially compared to CH flowers. The variations in expression pattern were concomitantly consistent with the floral organ divergence that generates CH, inCL, and CL flowers in extended photoperiods. The downregulation of these B-class MADS-box genes occurred during the late developmental stages was not possible to alter the organ identity of petals and stamens, but might result in organ size reduction and developmental abortion. This was further supported by the observation that the extent of reduction in organ size/number was not strictly equivalent to the decrease extent of B-class gene expression in these organs (Additional file 6: Table S3), indicating that the drop in expression should not be a result of the lower proportion of petal/stamen tissues in CL and inCL flowers to those in CH flowers. Nonetheless, the CH-CL transition, as an indirect result of the downregulation in the expression of these B-class genes, cannot be excluded. Given the postdevelopmental role of B-class MADS-box genes [24, 50–52], we therefore concluded that long daylight inhibited the development of petals and stamens by directly or indirectly inhibiting the expression of *VpTM6-1*, *VpTM6-2,* and *VpPI* genes to produce CL flowers.

B-class MADS-box genes, encoding bifunctional transcription factors, activate or repress a substantial number of downstream targets [24], thus acting as a context-dependent transcriptional switch that directs flower development: either floral organ identity, or postdevelopment. Since floral homeosis did not occur in the CH-CL transition, possible postdevelopmental mechanisms of B-class genes in *Viola* were further discussed. In *Arabidopsis*, *AP3* and PI positively regulate the expression of *SPOROCYTELESS/NOZZLE* (*SPL/NZZ*) and *NAP* (for *NAC-LIKE, ACTIVATED BY AP3/PI*, also known as *NO APICAL MERISTEM*) [24, 53]. *SPL/NZZ* is necessary for the formation of anther walls, and is required during the late stages of stamen development for microsporogenesis and consequent pollen formation [54–57]. On the other hand, *NAP* is involved in the transition between cell division and cell expansion phases during the growth of petals and stamens [53]. *AP3/PI* also suppresses certain downstream genes, such as *GNC* and *GNC-LIKE* (*GNL*), that encode two GATA-type zinc finger proteins, which in turn regulate sugar response and nitrate metabolism genes, thereby providing a link between organ development and nutrient sensing [58]. Short photoperiods induce the expression of *VGA20ox* and *VGA3ox*, which increases gibberellins that are also involved in CH-CL flower development in *V. pubescens* [34]. Gibberellins regulate the expression of specific floral genes in *Arabidopsis* such as the B-class MADS-box genes [59, 60]. Therefore, upon perceiving regulatory signals such as photoperiod (or gibberellins), we assumed that the differential expression of these *Viola* B-class MADS-box genes might influence dimorphic flower development by regulating homologous genes *SPL/NZZ*, *NAP*, and *GNC*, which in turn affects the growth and development of petals and stamens. However, further functional studies involving genetic manipulation of *V. philippica* should be conducted to verify this hypothesis.

### The novel role of B-class MADS-box genes in dimorphic flower development

B-class MADS-box genes include both *AP3* and *PI* lineages that evolved to play a primary role in petal and stamen development and in the establishment of stamen functionality [12–16, 18, 61]. Coding sequence variations that lead to different PPIs associated with these proteins play an essential role in this functional evolutionary process [15, 16], whereas altering its expression pattern facilitates the acquisition of additional functions, thereby leading to new floral morphologies [17–19, 25, 50–52]. The transcriptional regulation of these genes roughly includes the initiation of expression during early floral stages and the maintenance of expression through the majority of floral organ development [6, 9, 46–48, 62, 63]. When expression is affected during floral initiation, homeotic alterations usually occur, resulting in multiple corolla or calyces [7–9, 15, 64, 65]. However, when the expression is altered during late developmental stages, the maturation or function of the organs is affected [13–16, 24, 40–43]. Compared to previously identified B-class MADS-box genes [3–6], *VpTM6-1*, *VpTM6-2,* and *VpPI* in *V. philippica* shared a similar expression pattern during flower development, indicating that these genes may play a conserved role in establishing floral organ identity. However, we found that differential expression of these genes during late developmental stages of CH and CL flowers is regulated by variations in photoperiod, thereby suggesting a novel role for these B-class MADS-box genes in dimorphic flower development.

Floral morphology, such as petal size/shape instead of change of organ identity, could be regulated by artificial control of B-class gene expression, such as in *M. truncatula* [14] and tomato [13], whereas severely downregulating either *PFGLO2* or *PFTM6* only results in the production of immature pollen in *P. floridana* [15, 16]. Interestingly, regulating gene expression to generate distinct floral morphology also occurs in nature, such as in the decrease in B-class MADS-box genes to generate CL flowers in *V. philippica*. The molecular mechanism underlying this regulation of B-class MADS-box genes in response to photoperiods in petals and stamens requires further investigation. Currently, at least two levels of divergence have been observed. *VpTM6-1*, *VpTM6-2,* and *VpPI* are expressed at higher levels in petals than in stamens in CH flowers. However, this decrease in the level of expression was more extensive in petals than in stamens, correlating to the absence of petals and the reduction to two fertile stamens in CL flowers. Moreover, VpPI interacted strongly with VpTM6-1, but did weakly with VpTM6-2, which was indicative of *VpTM6-1* and *VpTM6-2* divergence in dimerization activity and transcriptional activity. Such differences in heterodimerization capabilities have been observed in species that harbor duplicated genes such as *Petunia* and *Physalis* [12, 15, 16]. Since a single amino acid change in the I doamin is sufficient to alter PI-like dimerization activity during maize domestication [66], that this type of divergence might result from extensive seuquence divergence of the two VpTM6 proteins, particularly the two deleterious alterations (K66M, and S63_T64insYV) in the I domain, should also be considered in understanding the functional divergence of B-class MADS-box genes in *V. philippica*.

## Conclusions

The comprehensive investigation of floral MADS-box genes could facilitate better understanding of CH and CL flower development in *V. philippica*. Nevertheless, to our knowledge, we were the first time to reveal that the differential floral expression of B-class MADS-box genes after floral induction and organogenesis in response to variations in photoperiod is associated with the development of the CH-CL breeding system in *Viola*. Our findings present new insight into the development and evolution of dimorphic flowers.

## Methods

### Plant materials and growth conditions

Seeds of *V. philippica* were collected and stored at the Northwest Normal University (Lanzhou, Gansu, China). They are available upon request. The seeds were sterilized by immersion in 30 % sodium hypochlorite solution for 20 min, and then rinsed three times with distilled water before sowing in triangular flasks containing MS (Murashige and Skoog) culture medium. After the fourth true leaves developed, the seedlings were transplanted to individual 100-mm (0.5 L) diameter plastic pots containing peat and vermiculite (v/v = 2:1). The plants were grown at 22-28 °C under 8-, 10-, 12-, 14-, and 16-h daylight, respectively, in a growth chamber. The temperature was kept by air-conditioners. The humidity was kept around 48 %, and light intensity was 116 μmol m^−2^s^−1^. The plants under different daylight were grown in parallel on the different shelves. The daylight length was controlled by time controller in each cultivated shelf. To avoid the reciprocal influence, each shelf was enclosed by black cloth that was manually removed every day10:00 am for 8 h for ventilation.

### Flower morphology observation

The morphology of flower buds was observed under a stereomicroscope (Olympus SZ61, Tokyo, Japan). The ratio of CH, inCL, and CL flowers was evaluated under 8-, 10-, 12-, 14-, and 16-h daylight, respectively. One hundred plants were observed in each photoperiod, and about 200 flower buds were counted in each case. To measure floral organ size, 10 mature flowers of each type (CH, inCL, and CL) were analyzed. The images were captured using a camera linked to a stereomicroscope (Olympus SZ61, Tokyo, Japan).

### Scanning electron microscopy and histological analyses

Flower buds at different stages were fixed in a 3:1 (v/v) ethanol:glacial acetic acid solution and kept at 4 °C, dehydrated through an increasing ethanol gradient of up to 100 % ethanol, and dried in a critical point drier. Samples were imaged using a scanning electron microscope (Hitachi S-450, Tokyo, Japan). For histological analyses, the whole green flower buds were dyed for 30 h using Love’s hematoxylin to dark red color. Then, the dyed flower buds were washed in running water for 5 h to remove excess dye until these became blue. Finally, the blue flower buds were dehydrated through an increasing ethanol gradient, cleared using xylene, and embedded in Paraplast (Sigma P3683, St. Louis, Missouri, USA). Cross sections (7-μm thickness) of the flower buds were mounted on the slides with water at 40 °C. The wax was cleared from the slides by washing with 100 % xylene. The images of the cleared slides were finally photographed under a Leica microscope (DMI4000B, Wetzlar, Germany).

### Sequence isolation

The cDNA fragments of the targeted genes were isolated using degenerate primers (Additional file 7: Table S4) that were designed based on the conserved regions of the *APETALA3 (AP3)* and *PISTILLATA (PI)* orthologs from various plant species in GenBank (http://www.ncbi.nlm.nih.gov/). The full-length cDNA was assembled using 3′/5′-rapid amplification of cDNA ends (RACE). Universal 3′ and 5′ PCR primers were supplied by the SMARTer™ cDNA Library Construction Kit (Clontech, Mountain View, California, USA). After the RACE experiments, the full-length cDNA was amplified by routine RT-PCR using gene-specific primers (Additional file 7: Table S4). The cycling program consisted of an initial denaturation at 94 °C for 5 min, followed by 35 cycles at 94 °C for 30 s, 55 °C (degenerate primers) or 68 °C (gene-specific primers) for 30 s, 72 °C for 30 s, and a final extension of 72 °C for 10 min.

### Sequence divergence evaluation and phylogenetic analysis

Neutral or deleterious amino acid mutations were predicted by PROVEAN, the protein variation effect analyzer with default parameter setting (http://provean.jcvi.org/index.php). The amino acid sequences of these genes were aligned using CLUSTALW1.81 under default settings with manual adjustments. Gaps were introduced for proper alignment. The neighbor-joining phylogeny trees using protein sequences were constructed using MEGA5 [67]. Bootstrap values were based on 1,000 replicates.

### qRT-PCR

The plant growth cycle of *V. philippica* was too long, few floral buds developed under 8-h daylight. Therefore, stages 3, 4, and 5 floral buds, young leaves, and floral organs of mature flowers were respectively collected under 10-, 12- and 16-h light period in the glasshouse. Total RNA was extracted using TRIzol reagent (TIANGEN, Beijing, China). For qRT-PCR analysis, a PrimeScript RT Reagent Kit (TaKaRa, Dalian, China), SYBR Premix EX Taq II (TaKaRa, Dalian, China), and gene-specific primers (Additional file 7: Table S4) were used. An 18S ribosomal RNA gene was used as internal reference (AB354544.1). The primers were designed using DNAMAN, and evaluating of their specificity and primer efficiency indicated that they were suitable for comparative qRT-PCR analysis (Additional file 8: Figure S4). The amplification conditions were 95 °C for 30 s for one cycle, followed by 40 cycles of 95 °C for 5 s, and 60 °C for 30 s. Three independent biological samples were used. Expression levels were calculated according to Livak and Schmittgen [68].

### RNA in situ hybridization

Floral buds at various developmental stages were fixed in 4 % (wt/vol = 4 g/100 mL) paraformaldehyde and embedded in Paraplast (Sigma P3683, St. Louis, Missouri, USA). When ATG was set as 1, around 356-bp fragment of *VpTM6-2* (positions 331–687) and *VpPI* (positions 236–591) was used as template for both sense and antisense probe synthesis using the DIG RNA labeling kit (Roche, Mannheim, Germany) and T7 RNA polymerase (Roche, Mannheim, Germany). Hybridization was performed as described by Javelle [69], with the alteration that we washed the slides at a temperature of 50 °C. Sections of floral tissues of CH, inCL and CL flowers were incubated in the same hybridization solution for each probe. Images were captured with a Leica microscope (DMI4000B, Wetzlar, Germany).

### Yeast two-hybrid analysis

The ORFs of *VpTM6-1*, *VpTM6-1,* and *VpPI* were cloned into the pGADT7 or PGBKT7 vector (Clontech, Mountain View, California, USA). The co-transformed yeast cells of BD and AD fusion plasmids were selected by growth on SD plates lacking leucine (Leu) and tryptophan (Trp). Interactions were analyzed on the SD plates lacking Leu, Trp, adenine (Ade), and histidine (His), and further confirmed by the non-lethal ß-galactosidase activity assay in the yeast strain AH109. Before checking PPIs, the toxicity and self-activation capability of these B-class MADS-domain proteins were checked. These experiments were performed according to Gong and He [70].

### Sequencing and primer information

Sequencing of all constructs and primer synthesis were performed by Huada (Beijing, China). All primers used in this study are presented in Additional file 7: (Table S4).

## Abbreviations

*AP3*, *APETALA3*; cDNA, complementary DNA; CH, chasmogamous; CL, cleistogamous. *GLO*, *GLOBOSA*; NJ, neighbor-joining; ORF, open reading frame; *PI*, *PISTILLATA*; PPI, protein-protein interaction; qRT-PCR, quantitative RT-PCR; RACE, rapid amplification of cDNA ends; SNP, single nucleotide polymorphism; *TM6*, *TOMATO MADS BOX GENE 6*

## Additional files

Additional file 1: Table S1. Protein sequence variation of AP3- (a) and PI-lineage (b) genes. (PDF 169 kb) Additional file 2: Table S2. Amino acid divergence between the two VpTM6 paralogs. (PDF 177 kb) Additional file 3: Figure S1. Multiple sequence alignment of B-class MADS-box genes. (PDF 312 kb) Additional file 4: Figure S2. Gene expression during floral organogenesis and development. (PDF 590 kb) Additional file 5: Figure S3. Protein-protein interactions in yeast. (PDF 180 kb) Additional file 6: Table S3. The reduction extent of organ size and gene expression in CH-CL transition. (PDF 336 kb) Additional file 7: Table S4. The primers used in the present study. (PDF 252 kb) Additional file 8: Figure S4. Primer evaluation for qRT-PCR. (PDF 342 kb)
