# Supplementary material for: Expression of B-class MADS-box genes in response to variations in photoperiod is associated with chasmogamous and cleistogamous flower development in Viola philippica
Source: BMC Plant Biol. 2016 Jul 7;16:151. doi: 10.1186/s12870-016-0832-2 (PMC4936093; doi:10.1186/s12870-016-0832-2)
Supplement: Additional file 1: Table S1. — Protein sequence variation of AP3- (a) and PI-lineage (b) genes. (PDF 169 kb) [file 12870_2016_832_MOESM1_ESM.pdf]

Table S1. Protein sequence variation of AP3- (a) and PI-lineage (b) genes.

a	LeDEF	PfDEF	NtDEF	PhDEF	AmDEF	MtNMH7	AtAP3	MtTM6	PhTM6	LeTM6	PfTM6	VpTM6-1	VpTM6-2	OsMADS16
VpTM6-1	57.6	59.7	61.4	59.7	61.4	53.9	53.7	62.3	66.8	62.2	64.8	100	92.6	54.0
VpTM6-2	55.5	59.4	61.4	60.3	60.1	53.7	51.1	62.0	64.6	59.4	63.4	92.6	100	53.6

b	LeGLO2	PfGLO1	PfGLO2	NtGLO	PhGLO1	PhGLO2	MtPI	MtNGL9	AmGLO	AtPI	VpPI	OsMADS2
VpPI	65.1	64.8	67.0	66.7	65.4	67.5	71.3	64.1	67.9	63.2	100	61.0
