# Supplementary material for: Expression of B-class MADS-box genes in response to variations in photoperiod is associated with chasmogamous and cleistogamous flower development in Viola philippica
Source: BMC Plant Biol. 2016 Jul 7;16:151. doi: 10.1186/s12870-016-0832-2 (PMC4936093; doi:10.1186/s12870-016-0832-2)
Supplement: Additional file 2: Table S2. — Amino acid divergence between the two VpTM6 paralogs. (PDF 177 kb) [file 12870_2016_832_MOESM2_ESM.pdf]

Table S2. Amino acid divergence between the two VpTM6 paralogs.

Amino acid substitution	PROVPROVEAN score	Amino acid property	Amino acid deletion / insertion
K10M	-1.792, Neutral	Basic to Nonpolar	
S50N	0.091, Neutral	Polar to Polar	
S61A	-2.000, Neutral	Polar to Nonpolar	S63_T64insYV (-10.595, Deleterious)<br>Y, Polar; V, Nonpolar
K66M	-3.735, Deleterious	Basic to Nonpolar	
T82A	-0.996, Neutral	Polar to Nonpolar	
R139K	2.751, Neutral	Basic to Basic	
C150Y	3.753, Neutral	Polar to Polar	
N182D	-2.321, Neutral	Polar to Acid	
F185A	-0.058, Neutral	Nonpolar to Nonpolar	
V190L	-0.001, Neutral	Nonpolar to Nonplar	
H182L	3.244, Neutral	Basic to Nonpolar	N207_H210del (-1.671, Neutral)<br>N, Polar; Q, Polar; H, Basic
E215D	0.478, Neutral	Basic to Basic	
R220G	3.155, Neutral	Acid to Polar	

PROVEAN Score was predicted by PROVEAN (http://provean.jcvi.org). The cutoff value was -2.5, when the score more than -2.5. the prediction was neutral; when the score was less than -2.5, the prediction was deleterious. S63_T64insYV, VpTM6-2 has Y and V while VpTM6-1 lose the two amino acids; N207_H210del, 4 amino acids from N at position 207 to H at position 210 in VpTM6-1 were deleted from VpTM6-2. The sequence alignment of the two VpTM6 proteins is presented in Figure 3A.
