# Supplementary material for: Expression of B-class MADS-box genes in response to variations in photoperiod is associated with chasmogamous and cleistogamous flower development in Viola philippica
Source: BMC Plant Biol. 2016 Jul 7;16:151. doi: 10.1186/s12870-016-0832-2 (PMC4936093; doi:10.1186/s12870-016-0832-2)
Supplement: Additional file 3: Figure S1. — Multiple sequence alignment of B-class MADS-box genes. (PDF 312 kb) [file 12870_2016_832_MOESM3_ESM.pdf]

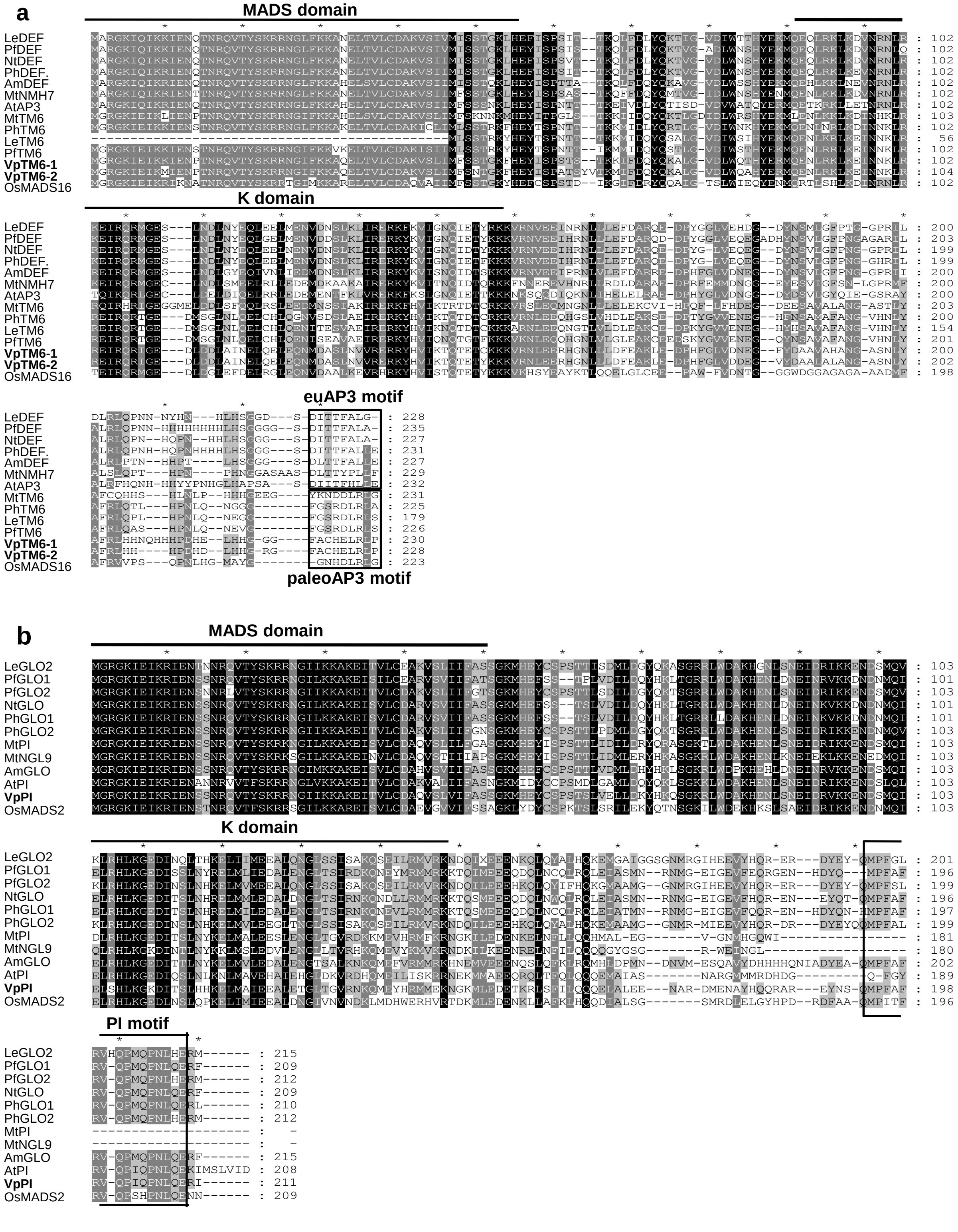


**Figure S1**. Multiple sequence alignment of B-class MADS-box genes.
**a** AP3-lineage. **b** PI-lineage. Gaps are introduced for proper alignment. The M, I, K, and C domains of each MADS-domain protein are indicated.
