# Supplementary material for: Expression of B-class MADS-box genes in response to variations in photoperiod is associated with chasmogamous and cleistogamous flower development in Viola philippica
Source: BMC Plant Biol. 2016 Jul 7;16:151. doi: 10.1186/s12870-016-0832-2 (PMC4936093; doi:10.1186/s12870-016-0832-2)
Supplement: Additional file 4: Figure S2. — Gene expression during floral organogenesis and development. (PDF 590 kb) [file 12870_2016_832_MOESM4_ESM.pdf]

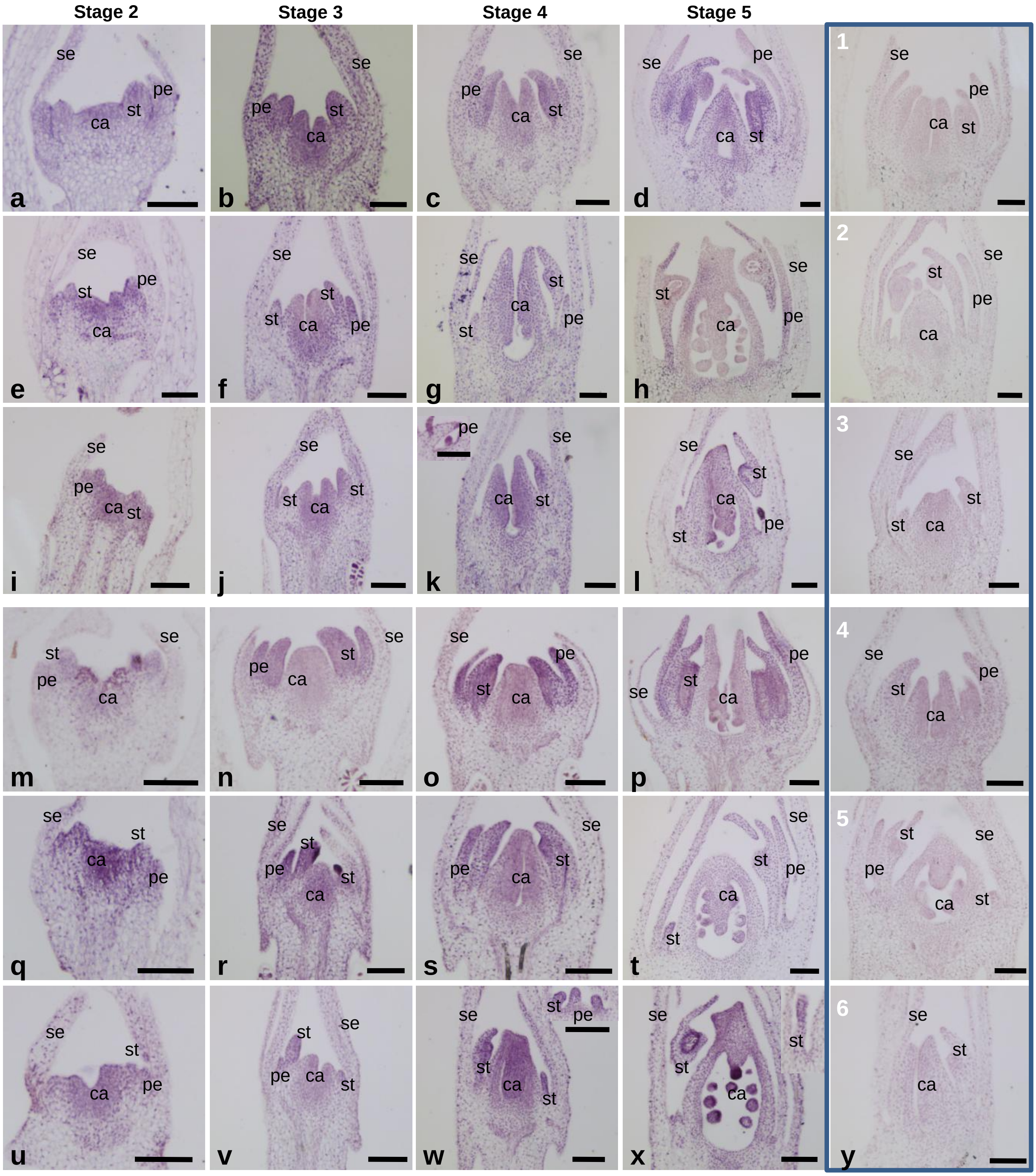

Figure S2. Gene expression during floral organogenesis and development.
a–l *VpTM6* (both *VpTM6-1* and *VpTM6-2*) expression during stages 2 to 5 of CH, inCL, and CL flowers. **a–d** CH flowers. **e–h** inCL flowers. **i–l** CL flowers. **m–x** The *VpPI* expression during stages 2 to 5 of CH, inCL, and CL flowers. **m-p** CH flowers. **q–t** inCL flowers. **u–x** CL flowers. Determination of gene expression levels was performed using *in situ* hybridization. The developmental stages are defined as that in Fig. 2. **y** Hybridization was performed using sense probes. The 1, 2, and 3 indicate *VpTM6*, whereas 4, 5, and 6 represent *VpPI*. Bars = 100 μm. se, sepal; pe, petal; st, stamen; ca, carpel.
