# Supplementary material for: Expression of B-class MADS-box genes in response to variations in photoperiod is associated with chasmogamous and cleistogamous flower development in Viola philippica
Source: BMC Plant Biol. 2016 Jul 7;16:151. doi: 10.1186/s12870-016-0832-2 (PMC4936093; doi:10.1186/s12870-016-0832-2)
Supplement: Additional file 5: Figure S3. — Protein-protein interactions in yeast. (PDF 180 kb) [file 12870_2016_832_MOESM5_ESM.pdf]

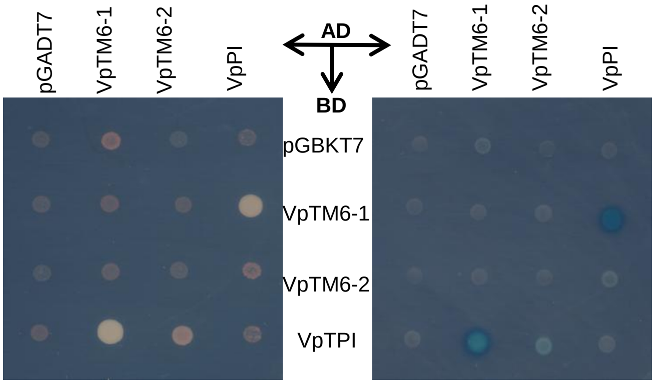

Figure S3. Protein-protein interactions in yeast.
Each of the obtained BD constructs was respectively transformed into the yeast strain AH109 and plated on SD/-Trp plates, and normal yeast growth indicated that these constructs could synthesize the engineered MADS-domain proteins without toxicity to yeast cells, while no cell growth was observed when the transformants were plated on SD/-Trp-His plates indicating that these proteins do not self-activate (Data not shown). And then PPIs were detected among the indicated B-class MADS-domain protein. The combination of the bait proteins (BD, vertical arrow) and the prey proteins (AD, horizontal arrows) is indicated. The left panel shows the growth of the co-transformed yeast cells on SD/Leu-Trp-His-Ade plates. The right panel shows the results of the non-lethal ß-galactosidase assay. The combinations of the MADS-box protein prey or bait construct with empty vector pGBKT7 or pGADT7 are used as controls.
