# Supplementary material for: Expression of B-class MADS-box genes in response to variations in photoperiod is associated with chasmogamous and cleistogamous flower development in Viola philippica
Source: BMC Plant Biol. 2016 Jul 7;16:151. doi: 10.1186/s12870-016-0832-2 (PMC4936093; doi:10.1186/s12870-016-0832-2)
Supplement: Additional file 6: Table S3. — The reduction extent of organ size and gene expression in CH-CL transition. (PDF 336 kb) [file 12870_2016_832_MOESM6_ESM.pdf]

Table S3. The reduction extent of organ size and gene expression in the CH-CL transition.

Flower type	The reduction ratio of stamen number	The reduction ratio of petal number	The reduction ratio of stamen length	The reduction ratio of petal length	The reduction ratio of *VpTM6-1* expression level					The reduction ratio of *VpTM6-2* expression level					The reduction ratio of *VpPI* expression level				
					Fl1	Fl2	Mf	S	P	Fl1	Fl2	Mf	S	P	Fl1	Fl2	Mf	S	P
inCL	0.55±0.28	0.74±0.36	0.58±0.05	0.95±0.02	0.70	0.84	0.71	0.70	0.92	0.67	0.85	0.82	0.71	0.92	0.87	0.86	0.68	0.66	0.86
CL	0.60±0.00	1.00±0.00	0.65±0.03	0.98±0.01	0.76	0.86	0.52	0.76	-	0.90	0.92	0.71	0.77	-	0.90	0.89	0.65	0.63	-

The reduction ratio of organ size / number in CL and inCL flowers is relative to CH flowers, at least 10 flowers were randomly analyzed in each flower type. The reduction ration of gene expression in different floral tissues is relative to these in CH flowers (Figure 6). Fl1, Fl1 and Mf was defined in Figure 6. S, stamens; P, petals. The – indicates no petal in CL flowers.
