# Supplementary material for: Expression of B-class MADS-box genes in response to variations in photoperiod is associated with chasmogamous and cleistogamous flower development in Viola philippica
Source: BMC Plant Biol. 2016 Jul 7;16:151. doi: 10.1186/s12870-016-0832-2 (PMC4936093; doi:10.1186/s12870-016-0832-2)
Supplement: Additional file 7: Table S4. — The primers used in the present study. (PDF 252 kb) [file 12870_2016_832_MOESM7_ESM.pdf]

Table S4. The primers used in the present study.

Function	Gene name	Sequence (5'-3')
Degenerate primers	*VpTM6*	CACYGTTCTYTGYGATGC<br>AGRAGAMGGTTTCCATGTCTTTC
	VpPI	AGCCCTTCWACTWCGYTGRTT<br>CACDCGGAARGMAAAWGGYA
5'-RACE PCR	*VpTM6*	TTCGTCGAAGTGGCTGGGCTGATA
	VpPI	ACAGCTCAACCAGCGAAGTAGAAGGACT
3' -RACE PCR	*VpTM6*	CCATGAGTATATCAGCCCAGCCACTTC
	VpPI	AGGATGAAACCAAGCGCCTCAGC
Full-length cDNA	*VpTM6-1*	CATGGGGAAGAAGAGAAGAGACCT<br>AGCGGCAGGCTAGCTTGTATTC
	VpTM6-2	CATGGGGAAGAAGAGAAGAGACCT<br>AGCGGCAGGCTAGCTTGTATTC
	VpPI	TCCCTTTGAATCTTCTATCTTCTT<br>TAGGCAATAGACCCATGAGGTAGA
qRT-PCR	*18SRNA*	AAGACGAACAACTGCGAAAGC<br>AGCAACATCCGCCAATCC
	VpTM6-1	GGATTGGTGGACAATGAGGGAT<br>CTCCATGGTGGAGCTCATGA
	VpTM6-2	GGGTTGGTGGACGATGAGGGAG<br>TTCCGTGGTGGAGATCATGG
	VpPI	GAGCTAAGTCACCTCAAAGGGAAG<br>AACACCAGTAAGACCAGTTTCAAGG
in situ hybridization	*VpTM6*	ATAGGGGAGGATTTGGATGACCTGTAATACGACTCACTATA<br>GGGTCAAGGAAGGCGGAGTTCGTG
	VpPI	TGTGGGATGCTAAACATGAGAACCTAATACGACTCACTATA<br>GGGGGAAAAAGGCATCTGGGAGTTAT
Yeast-two hybrid	*VpTM6-1*	AGCATATGGGTCGTGGAAAGATTGAG<br>AAGAATTCTCAAGGAAGGCGGAGTTCGTG
	VpTM6-2	AGCATATGGGTCGTGGAAAGATTGAG<br>AAGAATTCTCAAGGAAGGCGGAGTTCGTG
	VpPI	AGCATATGGGGAGAGGAAAGATTGAG<br>AAGGATCCTTAAATCCTTTCCTGCAGATTTGG
