# Supplementary material for: Expression of B-class MADS-box genes in response to variations in photoperiod is associated with chasmogamous and cleistogamous flower development in Viola philippica
Source: BMC Plant Biol. 2016 Jul 7;16:151. doi: 10.1186/s12870-016-0832-2 (PMC4936093; doi:10.1186/s12870-016-0832-2)
Supplement: Additional file 8: Figure S4. — Primer evaluation for qRT-PCR. (PDF 342 kb) [file 12870_2016_832_MOESM8_ESM.pdf]

a

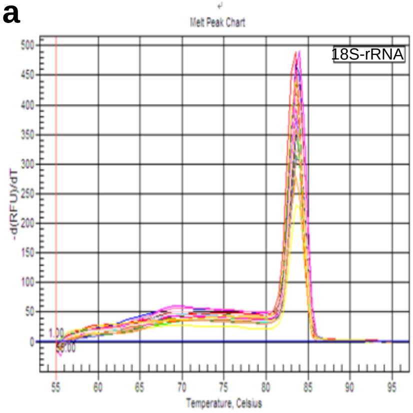

b

Gene / primer		GC%	Product length (bp)	Primer specificity	Primer efficiency
18S-RNA	F	48	136	√	100%
	R	56			
VpTM6-1	F	50	127	√	100%
	R	55			
VpTM6-2	F	63	115	√	100%
	R	55			
VpPI	F	50	96	√	100%
	R	44			

Figure S4. Primer evaluation for qRT-PCR.
a Melt curve peak chart of genes studied, using 18S rRNA gene as a representation. The occurrence of unique signal peak indicates that the primers are strongly gene-specific. The topology of melt curve peak charts of other genes was similar. **b** Summary of essential information of the primers for qRT-PCR. F, forward primer; R, reverse primer. √ indicates strong gene-specificity of the primers.
